# Association between knee alignment and knee pain in patients surgically treated for medial knee osteoarthritis by high tibial osteotomy. A one year follow-up study

**DOI:** 10.1186/1471-2474-10-154

**Published:** 2009-12-08

**Authors:** Annette W-Dahl, Sören Toksvig-Larsen, Ewa M Roos

**Affiliations:** 1 Department of Orthopedics, Clinical Sciences Lund, Lund University, Sweden; 2 Department of Orthopedics, Hässleholm Hospital, Hässleholm, Sweden; 3 Institute of Sports Science and Clinical Biomechanics, University of Southern Denmark, Odense, Denmark

## Abstract

**Background:**

The association between knee alignment and knee pain in knee osteoarthritis (OA) is unclear. High tibial osteotomy, a treatment option in knee OA, alters load from the affected to the unaffected compartment of the knee by correcting malalignment. This surgical procedure thus offers the possibility to study the cross-sectional and longitudinal association of alignment to pain. The aims were to study 1) the preoperative association of knee alignment to preoperative knee pain and 2) the association of change in knee alignment with surgery to change in knee pain over time in patients operated on for knee OA by high tibial osteotomy.

**Methods:**

182 patients (68% men) mean age 53 years (34 - 69) with varus alignment having tibial osteotomy by the hemicallotasis technique for medial knee OA were consecutively included. Knee alignment was assessed by the Hip-Knee-Ankle (HKA) angle from radiographs including the hip and ankle joints. Knee pain was measured by the subscale pain (0 - 100, worst to best scale) of the Knee injury and Osteoarthritis Outcome Score (KOOS) preoperatively and at one year follow-up. To estimate the association between knee alignment and knee pain multivariate regression analyses were used.

**Results:**

Mean preoperative varus alignment was 170 degrees (153 - 178) and mean preoperative KOOS pain was 42 points (3 - 86). There was no association between preoperative varus alignment and preoperative KOOS pain, crude analysis 0.02 points (95% CI -0.6 - 0.7) change in pain with every degree of HKA angle, adjusted analysis 0.3 points (95% CI -1.3 - 0.6).

The mean postoperative knee alignment was 184 degrees (171 - 185). The mean change in knee alignment was 13 degrees (0 - 30). The mean change in KOOS pain was 32 (-16 - 83). There was neither any association between change in knee alignment and change in KOOS pain over time, crude analysis 0.3 point (95% CI -0.6 - 1.2), adjusted analysis 0.4 points (95% CI 0.6 - 1.4).

**Conclusion:**

We found no association between knee alignment and knee pain in patients with knee OA indicating that alignment and pain are separate entities, and that the degree of preoperative malalignment is not a predictor of knee pain after high tibial osteotomy.

## Background

Varus and valgus malalignment are associated with medial and lateral knee osteoarthritis (OA) respectively. In natural history cohorts of knee OA, severity of malalignment has been shown to be associated with pain severity [1,2]. Additionally, frequent knee symptoms (i.e. pain, aching or stiffness on most days of the past month) was found to increase with increasing varus malalignment over 15 month [3]. In other studies malalignment was not associated with pain [4-6]. The relation of knee alignment and knee pain is thus still unclear and to our knowledge the association of alignment and pain has not previously been assessed in patients undergoing an intervention changing malalignment.

High tibial osteotomy (HTO) is a disease modifying intervention that reduces the tibiofemoral load in the damaged compartment of the knee joint. The purpose of HTO is to decrease malalignment, reduce pain, enhance function as well as delay or avoid the need of knee arthroplasty in younger and/or physically active patients with uni-compartmental knee OA. HTO offers the possibility to study the cross-sectional and longitudinal relation of knee alignment to knee pain.

Our aims were to study 1) the preoperative association of knee alignment determined as the Hip-Knee-Ankle (HKA) angle to preoperative knee pain and 2) the association of change in knee alignment with surgery to change in knee pain preoperatively compared to at one year postoperatively in patients operated on for knee OA by high tibial osteotomy using the hemicallotasis technique (HCO).

## Methods

### Patients

182 patients (68% men) mean age 53 year (range 34 - 69) scheduled for high tibial osteotomy (HTO) for medial knee OA, were consecutively included. The indication of surgery by the HCO is a consideration based on several aspects, as the presence of radiographic unicompartmental knee OA, knee alignment, pain, disability and level of activity both in working life and leisure time. When the orthopedic surgeon, in the present study one surgeon (STL) assessed all subjects, found an indication for HCO, the patient was given written and verbal information in a special outpatient clinic for patients treated by external fixation and the final decision on surgery was taken.

Of the 182 patients, 156 patients (86%) were available at the one-year follow-up. Fourteen patients did not return the questionnaire, two patients were revised to a total knee replacement, two patients had other surgeries, one patient had surgery in the contra lateral knee at time to follow-up and one patient had died.

### Radiographic assessment and classification of OA

Standing anteroposterior images of the knee were obtained in 15 degrees of flexion using a fluoroscopically positioned x-ray beam. Axial view of the patellofemoral joint was acquired with vertical beam and the subject standing with the knee in 50 degrees of flexion [7].

The Ahlbäck classification used for OA grading is based on reduction of joint space and the attenuation of subchondral bone. The classification includes 5 grades of radiographic knee OA; grade 1: joint space narrowing (<3 mm), grade 2: joint space obliteration, grade 3: minor bone attrition (<5 mm), grade 4: moderate bone attrition (5-10 mm) and grade 5: severe bone attrition (>10 mm) [8]. The radiographs were classified by one orthopaedic surgeon (STL). The Ahlbäck grade 1 corresponds approximately to Kellgren & Lawrence (K&L) [9] OA grade 2-3 (minimal to moderate, definite osteophyte, unimpaired joint space to moderate dimunition of joint space) and the Ahlbäck grade grade 2-5 to K&L OA grade 4 (severe, joint space greatly impaired with sclerosis of subchondral bone).

**Figure 1:**
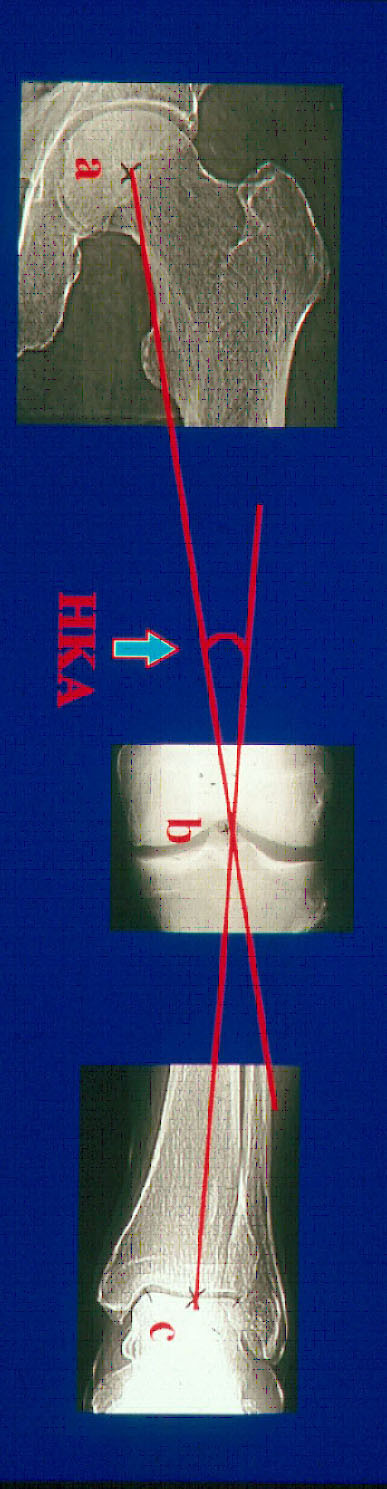
**Radiographic measurement of the Hip-Knee-Ankle angle (HKA-angle)**.

The preoperative knee alignment was assessed by the HKA angle. The HKA angle was obtained with the patient standing in a weight bearing position when radiographic anteroposterior and lateral views of the lower limb (hip, knee and foot) were taken. By drawing a line from the center of the femoral head to the midpoint of the tibial eminential spine and another line from this midpoint to the center of the talus surface of the ankle joint, the mechanical axis of the limb can be calculated [10]. The medial angle between the lines is the HKA angle (varus < 180°) (Figure 1). The accuracy and reproducibility of measurement of the HKA angle has been shown to be within 2 degrees [11]. In non-OA knees the mean HKA angle is 0.9-1.6 degrees in varus [12-14]. The HKA-angle was measured preoperatively as a part of the indication for surgery and postoperatively during the correction period to determine the progress of the correction and to determine that the desired alignment was obtained. The goal of correction is 4° valgus for the varus knee. Taking the reproducibility of HKA-angle measurement into account, 2 degrees is accepted as optimal correction. All patients were radiographically examined at the same radiographic department, the radiographs were taken by experienced technicians and the HKA angle was determined by radiologists with expertise in musculoskeletal radiology.

### Pain

Pain was measured by the subscale pain of the Knee injury and Osteoarthritisis Outcome Score (KOOS) preoperatively and at the 1 year follow-up [15]. KOOS is a 42-item self-administrated knee-specific questionnaire based on the WOMAC index [16]. KOOS was developed to be used for short-term and long-term follow-up studies of knee injury and knee OA. The KOOS comprises five subscales: pain, symptoms, activities of daily living function (ADL), sport and recreation function (Sport/Rec) and knee related quality of life (QOL). Standardized answer options are given (5 Likert boxes), and each respond is scored from 0 to 4. A percentage score from 0 to 100 is calculated for each subscale; 100 representing the best possible results. 8-10 points of the KOOS score is considered a clinically relevant difference [17]. The KOOS is previously used in HTO [18].

### Tibial osteotomy by the hemicallotasis technique (HCO)

**Figure 2:**
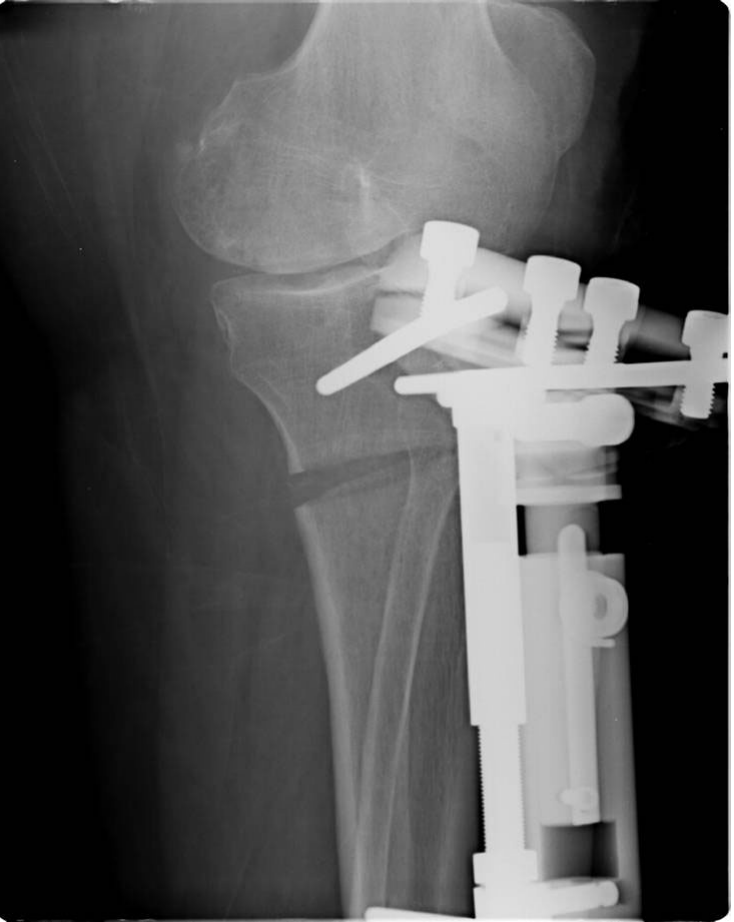
**Radiograph of high tibial osteotomy using the hemicallotasis technique**.

HCO is an open wedge osteotomy based on successive correction of the malalignment using an external fixation [18,19] (Figure 2).

### Statistics

The association between preoperative knee alignment (HKA angle) and preoperative knee pain (KOOS subscale pain), and change in knee alignment with surgery (the difference between preoperative HKA angle and postoperative HKA angle) and change in knee pain over time was assessed by simple regression analyses. Multiple regression analyses were used to control for potential confounding variables on preoperative KOOS pain (sex, age, Body Mass Index (BMI kg/m^2^), severity of knee OA (Ahlbäck grade 1-5) and preoperative knee alignment (HKA angle)) and on change in KOOS pain from preoperatively to the one year follow-up (sex, age, BMI, complications [septic arthritis, infection of the incision, DVT, replacement of pins, loss of correction and delayed healing], preoperative KOOS pain and change in knee alignment). The Ahlbäck grade 1 was used as reference and analyzed to Ahlbäck grade 2 and Ahlbäck grade ≥3 respectively (the category Ahlbäck grade ≥3 includes 13 patients with Ahlbäck grade 4 and one with Ahlbäck grade 5).

The results were presented with 95% confidence intervals (95% CI). P value < 0.05 was considered as statistically significant.

The study was approved by the Ethics Committee at the Medical Faculty, Lund University (LU-565-1) and was performed in accordance with the Declaration of Helsinki.

## Results

Patient characteristics for the 182 consecutive patients (mean age 52.8, 68% men) available at baseline and the 156 patients available at the one year follow up are given in table 1.

**Table 1:** Patient characteristics of the study group

	Preoperatively			1 year postoperatively		
	**All**	**Men**	**Women**	**All**	**Men**	**Women**

	n = 182	n = 123	n = 59	n = 156	n = 103	n = 53

**Age *year***						

mean	52.8	53.7	51	53.2	54.1	51.5

range	34-69	36-69	34-63	35-69	36-69	35-63

**BMI *kg/m2***						

Mean	28.9	28.8	29.3	29	28.7	29.6

Range	17.9-39.7	23-39	17.9-39.7	21-39.7	23-39	21-39.7

Ahlbäck OA grade *(n)#						

OA grade 1	30	18	12	26	16	10

OA grade 2	60	40	20	50	32	18

OA grade 3	71	50	21	66	45	21

OA grade 4	13	9	4	9	7	2

OA grade 5	5	1	0	1	1	0

**Preop HKA-angle**						

***Degrees***						

Mean	170.4	169.9	171.3	170.5	169.9	171.7

range	153-178	153-178	159-178	157-178	157-178	161-178

**KOOS Pain**						

Mean	42	47	38	42	45	37

Range	3-86	3-86	3-67	3-81	3-81	3-67

*Ahlbäck classification [8]

# missing data 7 patients

BMI = Body Mass Index

Pre op HKA angle = preoperativ HKA-angle

(<180 degree = varus)

### Preoperative cross-sectional analysis

Preoperatively, the mean HKA-angle was 170 degrees, i.e. on average the patients had 10° of varus alignment and the preoperative KOOS pain score was 42, (Table 1). There was no association between preoperative varus alignment and preoperative KOOS pain either crude or adjusted (Table 2).

**Table 2:** Relation of independent variables on preoperative pain and change in pain preoperatively to the one year follow-up

	Preoperative Pain			Change in Pain		
	Δ*	95% CI	p-value	Δ**	95% CI	p-value
Gender	-6.3	-12.6 - 0.1	0.06	-1.2	-9.6 - 7.1	0.8

Age	-0.7	-0.5 - 0.4	0.8	0.2	-0.4 - 0.8	0.5

BMI kg/m^2^	-1.1	-1.9 - -0.2	0.01	-0.8	-1.9 - 0.2	0.1

**OA grade#**						

grade 2	0.6	-7.9 - 9.2	0.9	-10.4	-21.5 - 0.7	0.07

grade ≥3	1.9	-6.6 - 10.4	0.7	-6.4	-17.2 - 4.3	0.2

Preoperative HKA-angle	0.3	-0.4 - 1	0.4			

Change in HKA-angle ##				0.4	-0.6 - 1.4	0.4

Complications				-1.4	-10.4 - 7.6	0.8

Preoperative Pain				-0.5	-0.7 - -0.3	<0.0001

* Estimated change of preoperative pain per unit change independent variable

** Estimated change of change in pain (preoperatively to the one year follow-up) per unit

change of independent variable

# OA grade according to the Ahlbäck (Ahl) classification, reference Ahl grade 1

## preoperative minus postoperative HKA-angle

### Longitudinal analysis

156 patients (86%) were available at the one year follow up (Table 1). The preferred correction (4 degrees valgus +/- 2 degrees) was obtained in 178/182 patients. The mean postoperative alignment was 184 degrees (range 171 - 185). The mean change in knee HKA-angle was 13 degrees (range 0 - 30). The mean change in KOOS pain was 32 points (range -16 - 83). There was no association between change in knee alignment with surgery and change in knee pain preoperatively to one year postoperatively either crude or adjusted (Table 2).

Preoperatively, higher BMI and female gender were associated with more pain.

More preoperative pain predicted less improvement in pain postoperatively and patients with Ahlbäck OA grade 2 tended to have less improvement in KOOS pain over time than patients with Ahlbäck OA grade 1 and 3 (Table 2).

**Figure 3:**
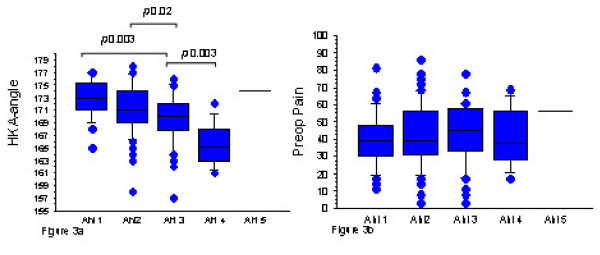
**Boxplot of preoperative HKA angle (a) and preoperative pain (b) for each Ahlbäck grade of knee OA (Median with quartiles)**. Any data observation which lays more than 1.5 IQR lower than the first quartile or higher than the third quartile is considered an outlier and marked as a dot. The horizontal line or "whisker" indicate where the smallest/highest value that is not an outlier by connecting it to the box).

Increasing OA grade was associated with more varus alignment. There was a statistically significant difference between the Ahlbäck categories of knee OA severity and preoperative HKA-angle (Figure 3a). However there was no association between Ahlbäck categories of knee OA severity and pain (Figure 3b).

## Discussion

We found no association between knee alignment and knee pain, neither preoperatively nor from preoperatively to one year postoperatively in patients operated on for medial knee OA by high tibial osteotomy using the hemicallotasis technique.

To our knowledge the association of alignment and pain has not previously been assessed in patients undergoing an intervention improving malalignment. The rationale for analysing this association is the belief that higher grade of preoperative HKA-angle may be related to less improvement in pain. However our results indicate that patients with more severe varus alignment experience similar pain relief from high tibial osteotomy by the hemicallotasis technique as patients with less varus alignment.

A strength of our study is the wide range of HKA angle and KOOS pain both preoperatively and over time. If there were any associations between preoperative HKA-angle and preoperative pain, or between change in pain and change in HKA-angle, the study had the possibility to detect them.

We used the Ahlbäck classification [8] to determine OA severity. The Ahlbäck classification, used especially in orthopedics and in northern Europe, primarily focus on reduction of the joint space as an indirect sign of cartilage loss while the more commonly used classification according to Kellgren & Lawrence takes osteophytes, joint space narrowing or both into account [9]. The Ahlbäck system differentiates between more severe grades of OA than the classification of Kellgren & Lawrence, which is useful in orthopedics and decisions relating to surgical treatment. The agreement between K&L grade 2-3 and Ahlbäck grade 1 as well as K&L grades 3-4 versus Ahlbäck grades 1-2 has been shown to be good (k 0.76 and 0.78) [20].

Our results differ from previous reported results on the relation of knee alignment and pain measuring alignment from long limb radiographs [1-3]. However our results are in line with results from studies measuring alignment from anteroposterior (AP) radiographs of the knee joint [4,5]. Reasons for the difference in results between studies may include the different populations, different methodologies for assessment of alignment and pain and interpretation of data.

### Different populations

In our study subjects about to have surgery for advanced OA were included which is in contrast to subjects recruited from the community with less advanced OA or at risk for knee OA [1-5]. However different study populations alone may not explain the difference as different methods were used.

### Assessment and interpretation of alignment

Different methods as well as different axis are used to determine the degree of deformity of the lower extremity. The mechanical axis by full-limb radiographic measures, the HKA-angle, is used in association with surgical interventions such as high tibial osteotomy and knee replacement. Knee alignment is sometimes determined from anteroposterior (AP) radiographs of the knee joint. This measure is however uncertain because the shorter images includes limited parts of the femur and tibia and makes it impossible to determine neither mechanical nor anatomical axis of the lower extremity.

Measurement of different angles, using AP and long leg radiographs respectively, the error in the measurement, and different definitions of normal, varus and valgus alignment may explain the contradictory results. Studies analysing the association of knee alignment to knee pain has not reported or discussed the possible error in the measurement of neither the anatomical axis nor the mechanical axis [2,4,5,21-23]. The technique, experience and accuracy of the performance of the radiographic examination are of importance to minimize the methodological error. Aspects that makes the measurement of alignment of the lower leg uncertain.

### Assessment and interpretation of pain

The mean KOOS pain score of 42 in this study is comparable to a preoperative score of 38 seen in patients having total knee replacement [24], indicating patients undergoing high tibial osteotomy having severe pain preoperatively. The mean improvement from high tibial osteotomy was 32 points at one year compared to 45 at one year after total knee replacement [24], indicating the effect of high tibial osteotomy being nearly as large as that from total knee replacement.

In previous studies the WOMAC [4,5] and the Visual Analogue Scale (VAS) [2] have been used as pain measures. Different pain instruments may be of minor importance as long as valid instruments are used and instrument-specific clinically relevant differences are considered. Sharma et al (2001) showed for example differences of 3.5 - 16 mm in pain assessed by the VAS between three different categories of varus alignment and an average VAS increase of 10 mm on a 0-100 mm scale in knee pain with each 5° of increased malalignment [2]. Clinically meningful differences in the Visual Analogue Scale (VAS) have been suggested to be 13-28 mm on a 100 mm scale depending on the initial VAS score [25].

In our study patients reported on average 1.5 KOOS points more pain on a 0-100 point scale per 5 degrees of varus alignment (Table 2). For the KOOS, an 8-10 point difference is considered a clinically relevant difference [17]. None of these studies showed clinically relevant differences with 5 degrees increasing malalignment, but the results were interpreted in opposite directions. Conclusions based on statistically significant results on the association of alignment to pain should be interpreted with caution if they are not clinically relevant.

In the cross sectional analysis preoperative pain was associated with increasing BMI while in the longitudinal analysis, there was no association. In the cross sectional analysis the change of preoperative pain per unit change of BMI was however negligible despite being significant. Patients with Ahlbäck grade 2 experienced clinically significant less improvement in pain over time compared to patients with Ahlbäck grade 1 but there was not a similar association for patients with Ahlbäck grade ≥3. This may reflect the well-known discordance between radiographic knee OA and symptoms [26].

## Conclusion

We found no association between knee alignment and knee pain in patients with knee OA indicating that alignment and pain are separate entities, and that the degree of preoperative malalignment is not a predictor of knee pain after surgery.

## Competing interests

The authors declare that they have no competing interests.

## Authors' contributions

AWD: study design, data selection, data analysis and preparation of manuscript. STL: data selection and preparation of manuscript. ER: study design, data analysis and preparation of manuscript. All authors read and approved the final manuscript.

## Pre-publication history

The pre-publication history for this paper can be accessed here:

http://www.biomedcentral.com/1471-2474/10/154/prepub
